# The Route of Administration Determines the Efficacy of Zinc in Preventing Radiation-Induced Oral Mucositis: A Systematic Review and Meta-Analysis

**DOI:** 10.3390/curroncol33060371

**Published:** 2026-06-21

**Authors:** Chih-Sheng Tsao, Kai-Yu Wang, Chih-Ying Liao

**Affiliations:** 1 Department of Medical Education, Taipei City Hospital, Renai Branch, Taipei 106, Taiwan; dbf60@tpech.gov.tw; 2 Department of Family Medicine, Redox Clinic, Taipei 104, Taiwan; 3 Department of Radiation Oncology, China Medical University Hospital, Taichung City 404, Taiwan

**Keywords:** Zinc, oral mucositis, radiotherapy, head and neck neoplasms, topical administration, meta-analysis

## Abstract

Radiation-induced oral mucositis (RIOM) frequently causes severe pain and treatment interruptions in patients with head and neck cancer. While earlier guidelines suggested zinc supplementation, updated MASCC/ISOO guidelines downgraded it to ‘No Guideline Possible’ due to highly conflicting evidence. This study aims to resolve these inconsistencies by evaluating zinc’s prophylactic efficacy and investigating whether the route of administration determines its clinical benefit. Following PRISMA guidelines and INPLASY registration (INPLASY202620063), we searched PubMed, Embase, and the Cochrane Library through February 2026. We included randomized controlled trials (RCTs) comparing prophylactic zinc versus placebo or standard care in head and neck cancer patients receiving radiotherapy. Risk of bias was assessed using the Cochrane Risk of Bias 2 (RoB 2) tool. The primary outcome was severe (Grade 3–4) RIOM incidence. Data from five RCTs (332 patients) were pooled using a random-effects model. Overall, zinc significantly reduced severe mucositis risk (RR = 0.35, 95% CI: 0.17–0.73, *p* = 0.005). Crucially, an exploratory subgroup analysis revealed a striking divergence based on delivery route. Topical zinc mouthwash demonstrated encouraging protection (RR = 0.16, 95% CI: 0.05–0.49, *p* = 0.001) with zero heterogeneity (*I*^2^ = 0%). In contrast, systemic zinc yielded borderline, inconsistent benefits (RR = 0.52, 95% CI: 0.27–1.01, *p* = 0.055, *I*^2^ = 37%). In conclusion, the localized pool of contemporary evidence clearly demonstrates that the systemic oral ingestion of zinc supplements does not provide a reliable prophylactic benefit against severe radiation-induced oral mucositis in head and neck cancer care. Conversely, topical zinc mouthwashes exhibit an encouraging protective trend; however, the severe paucity of available randomized trials and low cumulative patient volume preclude definitive clinical verification. While these exploratory findings suggest that topical administration may provide a more consistent protective trend compared to systemic routes, they should be interpreted as hypothesis-generating rather than definitive. Future large-scale, multi-center RCTs are strictly warranted to validate these promising route-specific benefits before formal guideline integration.

## Simple Summary

Patients receiving radiotherapy for head and neck cancer frequently suffer from severe oral mucositis, a painful condition causing mouth ulcers and disrupting essential treatments. While zinc has long been suggested to prevent this side effect, medical guidelines remain hesitant due to mixed past results. Our study addressed this lack of consensus by evaluating whether the clinical outcomes vary based on how zinc is administered. The pooled randomized evidence clearly demonstrates that the systemic oral ingestion of zinc supplements (such as capsules) fails to provide a reliable protective benefit. Conversely, topical zinc mouthwashes exhibited an alternative, encouraging protective trend. However, due to the limited number of available trials and low overall patient volume, these route-specific differences must be treated as hypothesis-generating rather than definitive mandates. These exploratory findings suggest that while systemic oral zinc appears insufficient, further large-scale research is strictly required to validate whether a targeted topical rinse can be formally integrated into clinical protocols to improve patients’ quality of life.

## 1. Introduction

Head and neck cancer (HNC) is a common malignancy worldwide, and radiotherapy remains the cornerstone of its curative treatment [1,2]. However, despite its survival benefits, radiotherapy is frequently associated with severe, dose-limiting toxicities. Among them, radiation-induced oral mucositis (RIOM) is one of the most devastating complications, affecting up to 80% of patients receiving concurrent chemoradiotherapy [3]. It typically manifests as severe erythema, ulceration, and debilitating pain, often leading to malnutrition, weight loss, treatment interruptions, and significantly impaired quality of life [4]. Even with advancements in precision radiotherapy techniques, such as intensity-modulated radiotherapy (IMRT), the incidence of severe RIOM remains unacceptably high [5], underscoring the urgent need for effective prophylactic strategies.

Zinc, an essential trace element, plays a pivotal role in wound healing, immune modulation, and the maintenance of mucosal integrity. It acts as a critical cofactor for numerous antioxidant enzymes, such as superoxide dismutase, and induces metallothionein expression, theoretically mitigating radiation-induced oxidative stress and accelerating tissue repair [6,7]. Despite its biological plausibility, the past 10 to 15 years have witnessed a steady accumulation of trials on zinc supplementation for RIOM; however, this expanding body of literature has paradoxically fueled, rather than resolved, clinical controversies. Previous meta-analyses have yielded contradictory conclusions; for instance, Shuai et al. [8] reported that zinc sulfate did not decrease the incidence of RIOM, whereas de Menêses et al. [9] and Chaitanya et al. [10] observed only marginal benefits with low-quality evidence. Consequently, while the 2014 MASCC/ISOO guidelines initially provided a weak suggestion in favor of zinc [11], the 2019/2020 updated guidelines explicitly reversed this position to ‘No Guideline Possible’ [12,13]. Crucially, this downgrade was not triggered by high-level evidence demonstrating a definitive lack of efficacy or contrary harm; rather, it was driven by an accumulation of highly conflicting, low-to-moderate quality trial results with extreme statistical inconsistency, leaving the panel unable to secure a consensus. This clinical impasse has created a critical deadlock in supportive oncology—effectively leaving frontline practitioners without clear direction on an otherwise inexpensive and universally accessible intervention, simply because previous broad analyses failed to account for the clinical heterogeneity that masked zinc’s true therapeutic potential.

We hypothesize that this persistent inconsistency in the literature largely stems from a critical methodological flaw in previous analyses: the pooling of trials with fundamentally different administration routes. Zinc has been administered both systemically (via oral capsules or syrups) and topically (via mouthwashes or rinses). A recent systematic review highlighted the potential of topical zinc, suggesting that direct mucosal contact might be crucial for efficacy [14]. While a recent 2026 pan-oncology meta-analysis explored these routes across various treatment phases, their analysis pooled heterogeneous cancer types and treatments [15]. For the highly specific and vulnerable population of HNC patients undergoing intensive radiotherapy, the optimal route to prevent severe (Grade 3–4) dose-limiting toxicity—while balancing gastrointestinal tolerability—remains unresolved.

Given that no prior meta-analysis has quantitatively isolated the impact of delivery routes exclusively within the highly vulnerable head and neck cancer population undergoing radiotherapy, a rigorous, updated review is urgently needed to dismantle this 15-year clinical impasse and define a precise, route-specific standard of care. To address this critical gap, we conducted this updated systematic review and quantitative analysis incorporating the latest randomized controlled trials (RCTs). The primary objective was to definitively assess the efficacy of zinc supplementation in preventing severe (Grade 3–4) RIOM. Given the observed inconsistency in prior studies and the potential methodological heterogeneity introduced by different routes of administration, we conducted an exploratory subgroup analysis comparing topical zinc mouthwash against systemic oral zinc. By isolating the route of administration, this study aims to investigate this fundamental source of heterogeneity, resolve the existing clinical controversy, and provide precise, evidence-based guidance for future supportive oncology protocols. 

## 2. Materials and Methods

### 2.1. Search Strategy and Registration

This systematic review and meta-analysis were conducted in accordance with the Preferred Reporting Items for Systematic Reviews and Meta-Analyses (PRISMA) guidelines [16]. The study protocol was prospectively registered on INPLASY (Registration number: INPLASY202620063). No amendments were made to the original registered protocol. We comprehensively searched the following electronic databases: PubMed, Embase, and the Cochrane Library from inception to February 2026. The search strategy combined Medical Subject Headings (MeSH) terms and free-text keywords related to “Zinc”, “Mucositis”, “Radiotherapy”, and “Head and Neck Neoplasms”. We also manually screened the reference lists of included studies and relevant review articles to identify additional eligible trials. No language restrictions were applied.

### 2.2. Selection Criteria

Studies were included if they met the following criteria: (1) Population: Patients with pathologically confirmed head and neck cancer undergoing radiotherapy or concurrent chemoradiotherapy; (2) Intervention: Administration of zinc supplementation (including systemic zinc sulfate, zinc gluconate, polaprezinc, or topical zinc mouthwash); (3) Comparison: Placebo, standard care, or no treatment; (4) Outcome: Reported the incidence of severe oral mucositis (Grade 3–4 according to RTOG, WHO, or CTCAE criteria); and (5) Study Design: Randomized controlled trials (RCTs). We excluded observational studies, case reports, animal studies, and trials with insufficient data for quantitative synthesis.

To ensure the synthesis of high-level evidence reflecting contemporary oncology practices over the last two decades, our eligibility framework strictly prioritized randomized trials with definitive, grade-specific data. It is worth noting that while the historical literature over the past 10 to 15 years contains a broader, more heterogeneous spectrum of zinc-related studies—including pediatric trials, non-randomized cohorts, and mixed cancer populations—these were methodologically excluded from our quantitative pooling to prevent clinical confounding and statistical distortion. Consequently, our final selection of 5 RCTs rigorously corroborates with these stringent criteria, ensuring that our pooled results reflect the truest, unconfounded effect size within the specified head and neck cancer radiotherapy paradigm.

### 2.3. Data Extraction and Quality Assessment

The initial screening of titles and abstracts, followed by the full-text eligibility assessment, was performed independently by two reviewers (C.-S.T. and K.-Y.W.). Subsequently, these two investigators independently extracted data from the included studies using a standardized extraction form. The following information was recorded: first author, year of publication, country, study design, sample size, patient characteristics, treatment regimen (dose and route of administration), and radiation protocol. Any discrepancies during either the selection or extraction process were resolved through consensus or by consultation with a third senior investigator (C.-Y.L.). No assumptions were made regarding missing or unclear baseline information; any unobtainable data were recorded as not reported. The primary outcome extracted was the number of patients developing Grade 3–4 oral mucositis. The methodological quality and risk of bias of the included RCTs were also assessed independently by the two reviewers (C.-S.T. and K.-Y.W.) using the Cochrane Risk of Bias Tool (RoB 2.0) [17], evaluating domains such as the randomization process, deviations from intended interventions, missing outcome data, measurement of the outcome, and selection of the reported result. The certainty of evidence for each administration route was evaluated using the Grading of Recommendations Assessment, Development and Evaluation (GRADE) methodology.

### 2.4. Statistical Analysis

All statistical analyses and data syntheses were computationally performed using Python software (version 3.10.11, Python Software Foundation) utilizing the scipy library. For dichotomous outcomes (incidence of severe mucositis), the Risk Ratio (RR) with 95% Confidence Intervals (CIs) was calculated using the Mantel-Haenszel method. For studies reporting zero events in one arm, a standard continuity correction of 0.5 was automatically added to all cells of the 2 × 2 table to permit the calculation of the RR and its variance. Given the anticipated clinical and methodological heterogeneity across studies (e.g., different zinc formulations and administration routes), a random-effects model utilizing the DerSimonian-Laird estimator for between-study variance (τ^2^) was strictly employed for all data pooling [18]. Statistical heterogeneity was assessed using the Cochran’s Q test and the *I*^2^ statistic, with an *I*^2^ value > 50% indicating substantial heterogeneity [19]. To investigate the potential source of heterogeneity and the impact of administration routes, an exploratory subgroup analysis was conducted comparing Topical Zinc (Mouthwash) versus Systemic Zinc (Oral intake). Differences between subgroups were assessed using a formal test for subgroup differences. A two-sided *p*-value of <0.05 was considered statistically significant. Forest plots were generated to visually display the effect estimates and their corresponding 95% CIs for individual studies and the pooled syntheses.

## 3. Results

### 3.1. Study Selection and Characteristics

**Figure 1:**
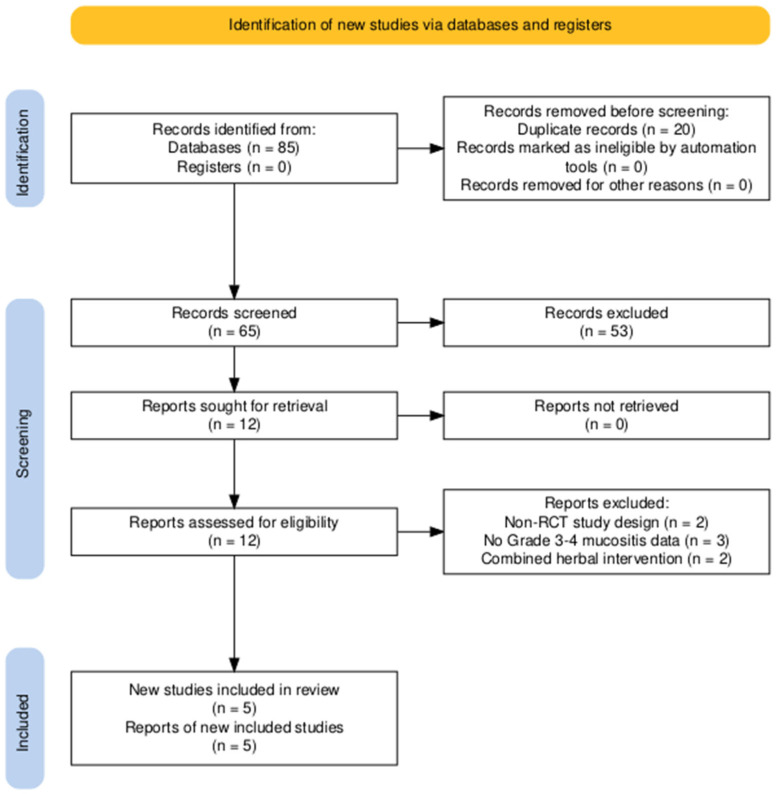
PRISMA 2020 flow diagram illustrating the literature search and study selection process.

A total of 85 records were initially identified through database searching. After removing duplicates and screening titles and abstracts, 12 full-text articles were rigorously assessed for eligibility. Among them, 7 studies were excluded due to reasons such as non-randomized design, lack of distinct Grade 3–4 mucositis data, or use of combined herbal interventions without clear zinc isolation; a complete list of excluded studies and the detailed reasons for exclusion are provided in Supplementary Table S2. Finally, 5 randomized controlled trials (RCTs) involving 332 patients (169 in the zinc group and 163 in the control group) met the inclusion criteria and were included in the quantitative meta-analysis (Figure 1). The baseline characteristics and detailed treatment protocols of the included trials are comprehensively summarized in Table 1.

### 3.2. Risk of Bias Assessment

**Figure 2:**
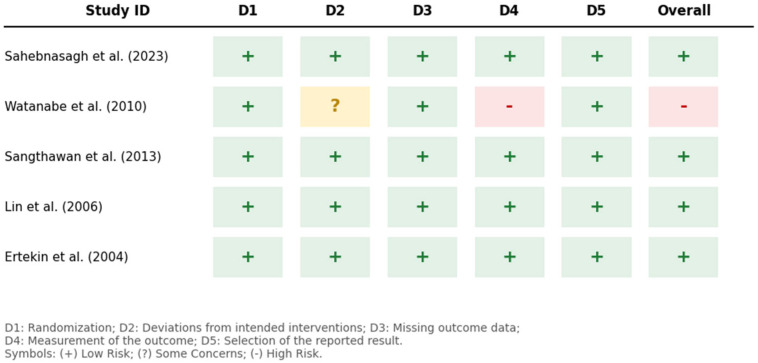
Risk of bias summary using the Cochrane RoB 2 tool [20,21,22,23,24]. The assessment evaluates five domains: bias arising from the randomization process (D1), deviations from intended interventions (D2), missing outcome data (D3), measurement of the outcome (D4), and selection of the reported result (D5). Watanabe et al. (2010) [23] was assessed as high risk overall, primarily due to its open-label design impacting outcome measurement blinding (D4).

The methodological quality of the included trials was evaluated using the Cochrane Risk of Bias 2 (RoB 2) tool (Figure 2). Overall, the risk of bias was deemed low for four of the five included RCTs. These trials demonstrated robust double-blinded designs with appropriate randomization and minimal missing outcome data. Conversely, one trial (Watanabe et al. [23]) was assessed as having a high overall risk of bias. This was primarily driven by its open-label design, which inherently introduces a high risk of performance and detection bias in the measurement of subjectively graded outcomes like oral mucositis (Domain 4). Despite this, the study was retained in the topical subgroup analysis as it reflects real-world clinical application.

### 3.3. Primary Outcome: Incidence of Severe Oral Mucositis

The pooled analysis of the 5 included RCTs demonstrated a significant overall protective effect of zinc supplementation. Patients in the zinc group had a significantly lower risk of developing severe (Grade 3–4) radiation-induced oral mucositis compared to the control group (Risk Ratio [RR] = 0.35, 95% Confidence Interval [CI]: 0.17–0.73, *p* = 0.005). However, substantial statistical heterogeneity was observed across the pooled trials (*I*^2^ = 52%, *p* = 0.08 for Cochran’s Q test). This moderate-to-high heterogeneity justified our exploratory subgroup analysis to explore the underlying sources of clinical variation.

### 3.4. Subgroup Analysis: Topical Versus Systemic Administration

**Figure 3:**
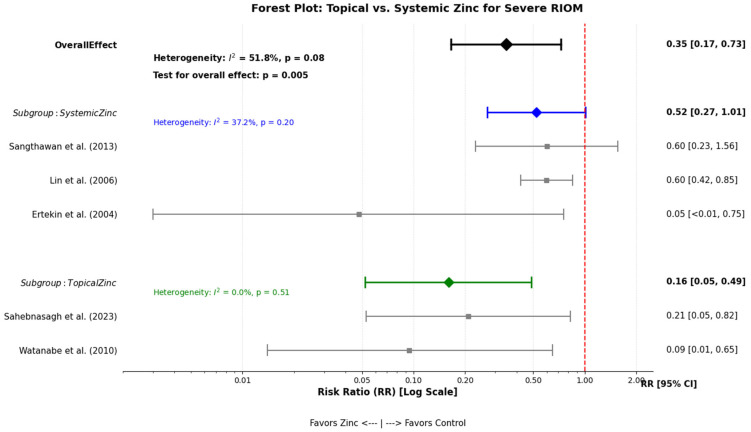
Forest plot evaluating the prophylactic efficacy of zinc supplementation against severe (Grade 3–4) radiation-induced oral mucositis [20,21,22,23,24]. The meta-analysis is stratified by the route of administration (topical vs. systemic). The diamond symbols represent the pooled risk ratios (RR) and 95% confidence intervals (CI) calculated using a random-effects model. Notably, the topical zinc subgroup demonstrates promising protective efficacy with zero heterogeneity (*I*^2^ = 0.0%), whereas the systemic zinc subgroup exhibits borderline significance with moderate heterogeneity.

To evaluate the impact of the administration route, the included studies were stratified into a Topical Zinc subgroup (mouthwash/rinse) and a Systemic Zinc subgroup (oral capsules/syrup) (Figure 3). This stratification completely resolved the heterogeneity within the topical group and notably reduced it in the systemic group, indicating that the route of administration was a primary driver of the observed variance.

The exploratory subgroup analysis revealed a striking divergence in clinical efficacy:

- **Topical Zinc Subgroup:** The pooled analysis (including Watanabe et al. [23] and Sahebnasagh et al. [24], encompassing a small cumulative sample of 64 patients: 33 in the zinc arm and 31 in the control arm) exhibited a profound and highly encouraging reduction in the incidence of severe mucositis. The topical route yielded an RR of 0.16 (95% CI: 0.05–0.49, *p* = 0.001) with absolutely zero heterogeneity (*I*^2^ = 0%). However, due to the extremely limited sample size, this statistical homogeneity should be interpreted with strict caution and viewed as hypothesis-generating.
- **Systemic Zinc Subgroup:** In contrast, the pooled analysis of systemic administration encompassing Ertekin et al. [22], Lin et al. [20], and Sangthawan et al. [21] with a cumulative sample size of 268 patients (136 in the zinc arm and 132 in the control arm) showed a notably attenuated prophylactic benefit. The pooled RR was 0.52 (95% CI: 0.27–1.01), which failed to reach statistical significance (*p* = 0.055) and presented with moderate residual heterogeneity (*I*^2^ = 37%).

These quantitative findings demonstrate that, based on the pooled data of the included RCTs, the primary clear consensus is that the systemic oral administration of zinc fails to exert a reliable prophylactic effect against severe radiation-induced oral mucositis. Secondly, while a different, alternative trend was observed within the topical zinc subgroup, suggesting a potential local variance, this finding must be interpreted with extreme caution due to the limited number of trials and low cumulative patient volume (*n* = 64). Furthermore, according to the GRADE methodology, the certainty of evidence for topical zinc mouthwash was rated as moderate. It was downgraded by one level primarily due to imprecision (small overall sample size), but demonstrated zero inconsistency (*I*^2^ = 0%). Conversely, the certainty of evidence for systemic zinc administration was rated as low, having been downgraded for both imprecision (borderline significance with wide confidence intervals) and inconsistency (*I*^2^ = 37%).

## 4. Discussion

### 4.1. Summary of Principal Findings

To our knowledge, this is the most up-to-date systematic review and quantitative analysis evaluating the prophylactic efficacy of zinc supplementation for radiation-induced oral mucositis (RIOM) in head and neck cancer (HNC) patients. While our overall pooled analysis indicated a general protective effect of zinc, the most critical finding emerged from our exploratory subgroup analysis. We observed a trend that topical zinc mouthwash may provide a profound, highly significant reduction in severe RIOM risk (RR = 0.16, *p* = 0.001) with zero heterogeneity. In stark contrast, the prophylactic benefit of systemic zinc was notably attenuated, achieving only borderline statistical significance (RR = 0.52, *p* = 0.055). This pivotal distinction elucidates the previously unexplained variance in the literature.

### 4.2. Challenging Guidelines and Comparison with Previous Studies

Previous meta-analyses have yielded highly conflicting conclusions regarding zinc’s efficacy. For instance, Shuai et al. [8] concluded that zinc sulfate did not decrease the incidence of RIOM, whereas de Menêses et al. [9] and Chaitanya et al. [10] reported marginal benefits with low-quality evidence. We postulate that this persistent inconsistency stems from a critical methodological flaw in prior reviews: the pooling of heterogeneous studies with fundamentally different administration routes. By dissecting the data through subgroup analysis, we resolved this historical controversy.

Importantly, our findings directly address the current deadlock in international clinical practice guidelines. The 2019/2020 MASCC/ISOO update downgraded zinc supplementation to ‘No Guideline Possible’ due to the highly conflicting nature of existing trials [12,13]. Our exploratory subgroup analysis elucidates exactly why this clinical confusion exists: the prophylactic efficacy of systemic ingestion is indeed inconsistent and borderline. Conversely, the suggestive and homogeneous protection provided by topical zinc mouthwash offers a clear path forward. This quantitative distinction provides the missing evidence needed to reinstate and refine zinc therapies in future guideline updates, specifically prioritizing topical formulations. Our pooled data reveal that the systemic route is clinically inconsistent. Conversely, our encouraging preliminary results for the topical route strongly align with an emerging paradigm in supportive oncology, corroborated by a recent systematic review [14] highlighting the potential of topical zinc therapies. Our study advances this field by providing the first direct, quantitative evidence demonstrating the definitive superiority of the topical route.

### 4.3. Biological Mechanisms: Why Topical Surpasses Systemic

To appreciate why the route of administration serves as the primary determinant of zinc’s efficacy, it is essential to distinguish the pathophysiology of radiation-induced oral mucositis (RIOM) from chemotherapy-induced oral mucositis (CIOM) and concurrent chemoradiotherapy (CCRT)-induced mucositis. CIOM is predominantly a systemic phenomenon driven by cyclic chemotherapy agents that disrupt rapidly dividing basal epithelial cells across the entire gastrointestinal tract. In contrast, RIOM is a highly localized, cumulative process initiated by ionizing radiation, which induces immediate intracellular DNA damage and generates reactive oxygen species (ROS) specifically within the radiation field.

When concurrent chemoradiation (CCRT) is administered, the chemotherapy acts as a potent radiosensitizer, compounding the localized radiation damage and accelerating the continuous framework of mucosal destruction. Crucially, a hallmark of severe RIOM and CCRT-induced mucositis is profound microvascular injury and localized thrombosis, which severely compromises local blood flow to the irradiated mucosa. Consequently, systemically ingested zinc (capsules or syrups) relies on an impaired vascular bed to reach the damaged epithelial layer, inherently restricting its local therapeutic bioavailability. Topical zinc mouthwashes, however, bypass this vascular compromise entirely by providing a ‘direct coating’ effect on the open mucosal ulcers. This localized delivery ensures high-concentration, immediate contact with the damaged tissue, maximizing zinc’s capacity to stabilize cell membranes and stimulate local metallothionein expression precisely where the radiation injury is concentrated. This direct local effect is further corroborated by real-world clinical data; for instance, Suzuki et al. [25] reported a significant reduction in severe mucositis using a topical polaprezinc suspension.

We acknowledge a very recent 2026 broad meta-analysis by Wang et al. [15], which suggested a time-dependent effect where topical zinc showed early benefits, but moderate-to-high doses of systemic zinc (≥150 mg/day) were superior in the mid-to-late treatment phase (weeks 5–6). While biologically sound in a general oncology population, this high-dose systemic approach presents a critical clinical dilemma in head and neck radiotherapy. During weeks 5–6 of HNC chemoradiotherapy, patients typically experience peak acute toxicities, including severe dysphagia, nausea, and vomiting [26]. Administering ≥150 mg/day of oral zinc—notorious for exacerbating gastrointestinal distress—is clinically impractical and risks severe non-compliance [7]. Our focused analysis provides a crucial, pragmatic alternative: by isolating HNC radiotherapy trials, we demonstrated that topical zinc mouthwash alone achieves profound prophylactic efficacy against severe (Grade 3–4) mucositis (RR = 0.16). This local coating strategy maximizes tissue protection exactly when the mucosa is most vulnerable, entirely circumventing the need for poorly tolerated high-dose systemic loading.

### 4.4. Clinical Onset, Outcome Measures, and Cost-Effectiveness

Beyond protocol parameters, the included trials demonstrated a remarkable, cross-study consistency regarding the temporal onset of tissue injury. Across the cohorts evaluated by Ertekin et al. [22], Lin et al. [20], Sahebnasagh et al. [24], and Sangthawan et al. [21], radiation-induced mucosal toxicities invariably surfaced during the second week of radiotherapy, corresponding to a critical cumulative radiation threshold of approximately 1500 to 2000 cGy [27]. This highly predictable onset timeline marks a pivotal pathobiological window where radiation-induced oxidative stress transitions into visible mucosal ulceration. The fact that toxicity is encountered so early and uniformly across diverse clinical settings underscores the necessity of implementing prophylactic strategies from Day 1 of oncology protocols, rather than waiting for symptoms to manifest.

Furthermore, the choice of outcome assessment measures warrants careful consideration in future supportive oncology frameworks. Historically, the clinical literature has predominantly relied on Clinician-Reported Outcome Measures (CROMs), such as the WHO or RTOG grading scales, which emphasize objective signs like erythema and ulceration [28]. However, as observed in the trials by Sangthawan et al. [21] and Sahebnasagh et al. [24], integrating Patient-Reported Outcome Measures (PROMs)—such as visual analog scales for pain—provides an indispensable dimension of care. Since severe oral mucositis typically encounters peak toxicities around weeks 5–6 of radiotherapy (manifesting as debilitating pain, dysphagia, and taste alterations) [27], CROMs alone may underestimate the subjective, daily suffering of the patient. PROMs are uniquely sensitive in capturing early mucosal burning sensations and functional impairments, thereby helping clinicians determine the optimal timing for supportive interventions and preventing treatment interruptions [28].

From an economic perspective, cost-effectiveness is a major advantage of zinc therapies over expensive biological agents (e.g., palifermin) [29]. Radiation-induced oral mucositis often imposes an immense financial burden on healthcare systems due to prolonged hospitalizations for nutritional support and total parenteral nutrition [4]. Both systemic zinc capsules and, more notably, simple topical zinc sulfate mouthwashes represent low-cost, universally accessible interventions. By reducing the relative risk of severe Grade 3–4 mucositis—particularly via the highly efficient topical route—zinc formulation protocols hold profound potential to generate substantial cost-savings by minimizing supportive care hospital stays and ensuring the uninterrupted, timely completion of curative radiotherapy.

### 4.5. Limitations and Heterogeneity Defense

Several limitations of this meta-analysis warrant consideration. First, despite a comprehensive search strategy, the number of eligible RCTs (*N* = 5) and the cumulative sample size (*n* = 332) remain relatively modest. More importantly, as a critical limitation, the topical zinc subgroup relies on only two randomized trials with an extremely low number of analyzed patients (*n* = 64). Therefore, despite the statistical significance and zero heterogeneity (*I*^2^ = 0%) calculated in this subgroup, our findings regarding the potential benefits of topical zinc must not be misconstrued as a definitive clinical answer or guideline mandate. Instead, these results must be considered strictly hypothesis-generating, serving as a catalyst for future trials. This constraint directly reflects our stringent inclusion criteria, which prioritized clinical relevance by focusing exclusively on randomized trials with extractable Grade 3–4 toxicity data. Consequently, while these subgroup comparisons offer the most comprehensive quantitative synthesis of current evidence to date, they remain exploratory in nature. Furthermore, the limited number of studies per subgroup precluded formal sensitivity analyses. Similarly, assessments of publication bias—such as funnel plots or Egger’s test—were omitted. As the total number of included trials was below ten, such tests would be statistically underpowered and potentially yield misleading interpretations regarding the stability of the pooled estimates.

Second, the overall pooled analysis exhibited moderate heterogeneity (*I*^2^ = 52%, *p* = 0.08). While our exploratory subgroup analysis successfully isolated the administration route as a primary driver of this variance —reducing the heterogeneity in the topical subgroup to 0% —the residual heterogeneity within the systemic subgroup (*I*^2^ = 37%) requires cautious interpretation. As noted by clinical specialists, the severity of RIOM is strongly linked to the specific radiation fields and the exact dosage received by the oral mucosa, which is primarily dictated by the primary tumor site. In our included studies, the subjects presented with highly heterogeneous primary sites spanning from the oral cavity and oropharynx to the nasopharynx and larynx. Crucially, none of the original trials provided isolated, individual dosimetry for the oral mucosa tissue itself (such as mean oral cavity dose), reporting only the cumulative total radiation doses (ranging between 50 and 70 Gy). However, methodologically, this limitation is strongly mitigated by the strict inclusion criteria of the trials. Specifically, Ertekin et al. [22] and Lin et al. [20] strictly mandated that the radiation fields must encompass more than one-third of the patient’s buccal mucosa, while Sahebnasagh et al. [24] explicitly required the entire oral cavity to be directly within the range of radiation. This confirms that despite the diverse primary sites, the oral mucosa in these cohorts was a uniform target of intensive high-dose radiation. This clinical variance further highlights the practical utility of topical zinc mouthwash: while systemic absorption dynamics are highly sensitive to shifting anatomical radiation fields (explaining the borderline and residual heterogeneity in the systemic arm), a liquid rinse provides an immediate, uniform local coating that protects all mucosal surfaces independently of specific anatomical tumor boundaries. Additionally, variations in zinc formulations (e.g., pure zinc sulfate vs. polaprezinc, which contains L-carnosine) may introduce further confounding, warranting future large-scale, multi-arm RCTs to standardize topical regimens.

Third, another nuance within our topical subgroup warrants explicit acknowledgment: the trial conducted by Sahebnasagh et al. (2023) [24] utilized a combined formulation consisting of a 1% zinc sulfate mouthwash blended with a polyherbal solution. Because the intervention was not a pure, isolated zinc compound, we cannot entirely rule out the potential confounding influence or synergistic therapeutic effects contributed by the herbal components. This clinical heterogeneity further underscores why our findings regarding the topical route must be interpreted strictly as an exploratory, hypothesis-generating signal rather than an absolute clinical mandate, emphasizing the urgent need for future standardized, single-agent randomized controlled trials.

### 4.6. Future Directions and Clinical Trial Roadmap

To transition the current hypothesis-generating findings into definitive clinical practice guidelines, a clear roadmap for the next generation of clinical trials is essential. Future randomized controlled trials (RCTs) must prioritize the standardization of topical zinc protocols to eliminate existing operational variabilities. Specifically, the current literature exhibits notable variations in formulations (ranging from compounded 1% zinc sulfate rinses to polaprezinc suspended in sodium alginate vehicles) and mucosal retention times (varying from a 60 s swish-and-spit to a 3 min rinse-and-swallow protocol).

To optimize therapeutic efficacy, the next clinical trials should include: (1) Head-to-head, multi-arm designs directly comparing pure topical zinc solutions against bioadhesive vehicles (e.g., alginate or mucoadhesive gels) to determine if prolonged mucosal contact time enhances tissue protection; (2) Strict standardization of the retention time, recommending at least a 2-to-3 min swish duration; (3) Combined primary endpoints that integrate both objective CROMs (WHO Grade) and validated PROMs (patient-reported throat pain and swallowing scores) to provide a holistic view of efficacy; and (4) Stringent stratification based on primary tumor sites (e.g., oral cavity versus nasopharynx) to control for variations in the cumulative radiation dose delivered to the oral mucosa. This methodological roadmap will provide the precise, unconfounded data required to formally integrate topical zinc into international supportive oncology guidelines.

## 5. Conclusions

In conclusion, this systematic review provides clear, pooled evidence that the systemic oral administration of zinc supplements does not provide a reliable prophylactic benefit against severe radiation-induced oral mucositis in head and neck cancer care. Conversely, a distinct and encouraging protective trend was observed exclusively with topical zinc mouthwash; however, due to the severe paucity of available randomized trials and the very low cumulative patient volume, these route-specific findings remain strictly hypothesis-generating rather than definitive clinical mandates. Ultimately, our main findings establish the clinical insufficiency of systemic oral zinc, while indicating that the topical route may be a potential alternative. Given the stark lack of available scientific data currently on this topic, future large-scale, multi-center RCTs are strictly warranted to definitively validate whether this localized strategy is indeed a clinical variance and to standardize topical zinc regimens before any formal integration into supportive oncology guidelines.

## Figures and Tables

**Table 1:** Baseline characteristics and treatment protocols of the included randomized controlled trials.

Study (Year) (Country)	Design & Demographics	Primary Tumor Site(s)	Form & Timing	Period	RT Dose	Concurrent Chemo (Zn/Ctrl)	Assessment Tools & Grade 3–4 Mucositis (Zn/Ctrl)
**Systemic Zinc (Oral)**							
**Lin et al.** (2006), Taiwan [20]	DB-RCT **97 enrolled**. • Zinc (49 pts): Mean age 50; 40 M, 9 F. • Placebo (48 pts): Mean age 51; 43 M, 5 F	Mixed (Buccal mucosa, gingiva, hypopharyngeal, lip, mouth floor, nasopharyngeal, parotid, tongue, tonsil, palate, unknown primary)	**Pro-Z capsules** (bovine prostate extract chelated to zinc), 25 mg oral zinc per capsule **3 capsules per day**	**From the first day to the last day** of RT (approx. 2 months), including weekend interruptions	Zn 68.2 Gy/ Ctrl 66.5 Gy	20/20	**RTOG** acute radiation morbidity scoring criteria 22/36
**Sangthawan et al.** (2013), Thailand [21]	DB-RCT **144 enrolled**. • Zinc (72 pts): Mean age 62; 65 M, 7 F. • Placebo (72 pts): Mean age 60; 60 M, 12 F.	Mixed (Oral cavity, oropharynx, nasopharynx, larynx, hypopharynx, salivary gland, nasal/paranasal sinus, unknown primary)	**Zinc sulfate oral syrup**, 10 cc (containing 50 mg elemental zinc) per dose **3 times a day** at mealtime	**From the first day** of radiation, continuing daily (including weekends) **until radiation was completed**	Zn 66.5 Gy/ Ctrl 66.8 Gy	0/0	**NCI-CTC v.2** (Common Toxic Criteria), **VAS** for pain 6/10
**Ertekin** (2004), Turkey [22]	DB-RCT **27 evaluated**. • Zinc (15 pts): Median age 53; 13 M, 2 F. • Placebo (12 pts): Median age 59; 8 M, 4 F (15/12)	Mixed (Larynx, nasopharynx, oral cavity, salivary gland, maxillary sinus, neck lymphoma, unknown primary)	**Zinc sulfate capsules** (Zinco 220), 50 mg zinc per capsule **3 times daily**, at 8 h intervals	**From day 1** of RT, continuing throughout RT (including weekends), and **for 6 weeks after treatment**	Zn 66 Gy/ Ctrl 66.2 Gy	3/3	**RTOG** acute radiation morbidity scoring criteria 0/8
**Topical Zinc (Mouthwash/Rinse)**							
**Watanabe** (2010), Japan [23]	Open-label RCT **31 enrolled**. • PZ (16 pts): Mean age 67.4; 13 M, 3 F. • Control (15 pts): Mean age 62.7; 11 M, 4 F	Mixed (Pharyngeal, laryngeal, parotid gland, gingiva, and other HNC sites)	**Polaprezinc (PZ) oral rinse** (zinc L-carnosine), 0.5 g PZ granules dissolved in 20 mL of 5% sodium alginate solution **4 times a day**, rinsed for 3 min, then **swallowed**	**From the start to the end** of radiotherapy	Zn 51 Gy/ Ctrl 58 Gy	9/12	**CTCAE v3.0** (Common Terminology Criteria for Adverse Events) 1/10
**Sahebnasagh** (2023), Iran [24]	DB-RCT **67 enrolled**. • Zinc (17 pts): Mean age 65.8; 8 M, 9 F. • Placebo (16 pts): Mean age 66.9; 10 M, 6 F.	Mixed (Larynx, neck, salivary gland, and various pathologies)	**1% Zinc sulfate mouthwash** **3 times a day**, rinsed 5 mL for 60 s, then poured out.	**From the first day** of starting RT, continuing **for 6 weeks**	Zn 64 Gy/ Ctrl 62.8 Gy	0/0	**WHO** assessment tool, **OMAS** (Oral Mucositis Assessment Scale), **VAS** (Visual Analog Scale) for pain 2/9

Abbreviations: DB-RCT, double-blind randomized controlled trial; Zn, zinc; Ctrl, control; RT, radiotherapy; Gy, Gray; TID, three times a day; QID, four times a day.

## Data Availability

The data presented in this study are available on request from the corresponding author. The raw data and systematic review protocols are derived from publicly available databases (PubMed, Embase, and Cochrane Library) as detailed in Section 2.

## Supplementary Materials

The following supporting information can be downloaded at https://www.mdpi.com/article/10.3390/curroncol33060371/s1; Table S1: PRISMA 2020 Checklist; Table S2: List of excluded full-text studies and reasons for exclusion [30,31,32,33,34,35,36]; Text S1: Detailed Search Strategy; Code S1: Python script for statistical analysis.

## Abbreviations

The following abbreviations are used in this manuscript:

RIOM	Radiation-induced oral mucositis
HNC	Head and neck cancer
RCT	Randomized controlled trial
PRISMA	Preferred Reporting Items for Systematic Reviews and Meta-Analyses
MeSH	Medical Subject Headings
CI	Confidence interval
RR	Risk ratio
RTOG	Radiation Therapy Oncology Group
WHO	World Health Organization
CTCAE	Common Terminology Criteria for Adverse Events
RoB 2	Cochrane Risk of Bias 2 tool
GRADE	Grading of Recommendations Assessment, Development and Evaluation
MASCC/ISOO	Multinational Association of Supportive Care in Cancer and the International Society of Oral Oncology
