# Supplementary material for: The Route of Administration Determines the Efficacy of Zinc in Preventing Radiation-Induced Oral Mucositis: A Systematic Review and Meta-Analysis
Source: Curr Oncol. 2026 Jun 21;33(6):371. doi: 10.3390/curroncol33060371 (PMC13298057; doi:10.3390/curroncol33060371)
Supplement: Supplementary file 1 [file curroncol-33-00371-s001.zip › Table S2, Text S1, Code S1.pdf]

Table S2. List of excluded full-text studies and reasons for exclusion

Study ID (Author, Year)	Reason for Exclusion
Ho, 2017 [30]	Non-randomized study design
Shuai, 2018 [31]	Non-randomized study design
Chaitanya, 2020 [32]	Use of combined interventions (e.g., honey/herbs) without clear zinc isolation
Muthukrishnan, 2017 [33]	Use of combined interventions (e.g., honey/herbs) without clear zinc isolation
Mosalaei, 2010 [34]	Lack of distinct Grade 3–4 mucositis data
Moslemi, 2014 [35]	Lack of distinct Grade 3–4 mucositis data
Lin, 2010 [36]	Lack of distinct Grade 3–4 mucositis data

Reference

30. Ho, J.K.H.; Choi, W.S. Prevention and Treatment of Oral Mucositis Caused by Chemo- and Radiotherapy in Head and Neck Cancer Patient. *Int. J. Oral Maxillofac. Surg.* **2017**, *46*, 144, doi:10.1016/j.ijom.2017.02.497.
31. Shuai, T.; Yi, L.J.; Tian, X.; Chen, W.Q.; Chen, H.; Li, X.E. Prophylaxis with Oral Zinc Sulfate against Radiation-Induced Oropharyngeal Mucositis in Patients with Head and Neck Cancer: Protocol for a Meta-Analysis of Randomized Controlled Trials. *Medicine* **2018**, *97*, doi:10.1097/MD.0000000000013310.
32. Chaitanya, N.; Badam, R.; Srikanth Aryasri, A.; Pallarla, S.; Garlpati, K.; Soni, P.; Gali, S.; Inamdar, P.; Parinita, B.; Zaheer, K.; et al. Efficacy of Improvised Topical Zinc (1%) Ora-Base on Oral Mucositis during Cancer Chemo-Radiation-A Randomized Study. *J Nutr Sci Vitaminol* **2020**, *66*, 93–97.
33. MUTHUKRISHNAN, A.; SHANMUGHAPRIYA, G. CHITOSAN IN THE TREATMENT OF RADIOTHERAPY INDUCED ORAL MUCOSITIS IN HEAD AND NECK CANCER PATIENTS: A RANDOMISD CLINICAL TRIAL. *Oral Surg. Oral Med. Oral Pathol. Oral Radiol.* **2017**, *124*, e198–e199, doi:10.1016/j.oooo.2017.05.502.
34. Lin, Y.S.; Lin, L.C.; Lin, S.W.; Chang, C.P. Discrepancy of the Effects of Zinc Supplementation on the Prevention of Radiotherapy-Induced Mucositis between Patients with Nasopharyngeal Carcinoma and Those with Oral Cancers: Subgroup Analysis of a Double-Blind, Randomized Study. *Nutr. Cancer* **2010**, *62*, 682–691, doi:10.1080/01635581003605532.
35. Mosalaei, A.; Nasrolahi, H.; Shafizad, A.; Ahmadloo, N.; Ansari, M.; Mosleh-Shirazi, M.A.; Mohammadianpanah, M. Effect of Oral Zinc Sulphate in Prevention of Radiation Induced Oropharyngeal Mucositis During and After Radiotherapy in Patients with Head and Neck Cancers. *Middle East J. Cancer* **2010**, *1*, 69–76.
36. Moslemi, D.; Babaee, N.; Damavandi, M.; Pourghasem, M.; Moghadamnia, A.A. Oral Zinc Sulphate and Prevention of Radiation-Induced Oropharyngeal Mucositis in Patients with Head and Neck Cancers: A Double Blind, Randomized Controlled Clinical Trial. *Int. J. Radiat. Res.* **2014**, *12*, 235–241.

Text S1. Detailed Search Strategy

Databases: PubMed (MEDLINE), Embase, and The Cochrane Library (CENTRAL). Search Date: Up to February 2026. Filters: Randomized Controlled Trials; Human subjects; English language.

1. PubMed Search Strategy

Step	Search Terms (Keywords & MeSH)
	(Population: Head and Neck Cancer & Radiotherapy)
#1	"Head and Neck Neoplasms"[MeSH] OR "head and neck cancer" OR "HNC" OR "radiotherapy"[MeSH] OR "radiation therapy" OR "irradiation"
	(Intervention: Zinc)
#2	"Zinc"[MeSH] OR "Zinc Compounds"[MeSH] OR "zinc" OR "zinc sulfate" OR "zinc gluconate" OR "polaprezinc" OR "Z-103"

Step	Search Terms (Keywords & MeSH)
	(Outcome: Oral Mucositis)
#3	"Stomatitis"[MeSH] OR "mucositis" OR "oral mucositis" OR "radiation-induced oral mucositis" OR "RIOM" OR "stomatitis"
	(Study Design: RCT Filter)
#4	"Randomized Controlled Trial"[Publication Type] OR "randomized controlled trial" OR "controlled clinical trial" OR "randomized" OR "placebo" OR "clinical trials as topic"[MeSH]
	(Final Combination)
#5	#1 AND #2 AND #3 AND #4

2. Embase Search Strategy (Example String)
('head and neck oncology'/exp OR 'head and neck cancer') AND ('zinc'/exp OR 'zinc sulfate' OR 'polaprezinc') AND ('mucositis'/exp OR 'oral mucositis') AND ('randomized controlled trial'/exp OR 'randomization')

3. Cochrane Library Search Strategy
#1 MeSH descriptor: [Head and Neck Neoplasms] explode all trees
#2 "head and neck cancer" OR "radiotherapy":ti,ab,kw
#3 MeSH descriptor: [Zinc] explode all trees
#4 "zinc" OR "zinc sulfate" OR "polaprezinc":ti,ab,kw
#5 MeSH descriptor: [Stomatitis] explode all trees
#6 "oral mucositis":ti,ab,kw
#7 #1 OR #2
#8 #3 OR #4
#9 #5 OR #6 #
10 #7 AND #8 AND #9

Code S1. Python script for the meta-analysis.

```
import numpy as np
import pandas as pd
import scipy.stats as stats

def calculate_meta_analysis(df, group_name="Overall"):
    print(f"=== {group_name} Meta-Analysis ===")

    # 1. Extract data
    study_names = df['Study'].values
    a = df['Zinc_Events'].values
    n1 = df['Zinc_Total'].values
    c = df['Control_Events'].values
    n2 = df['Control_Total'].values

    # 2. Continuity Correction for zero events
    # Apply standard 0.5 correction to all cells if any cell is 0 to avoid log(0)
    zero_mask = (a == 0) | (n1 - a == 0) | (c == 0) | (n2 - c == 0)
    a = np.where(zero_mask, a + 0.5, a)
    n1 = np.where(zero_mask, n1 + 1.0, n1)
    c = np.where(zero_mask, c + 0.5, c)
```

```
    n2 = np.where(zero_mask, n2 + 1.0, n2)

    # 3. Calculate individual study log(RR) and variance
    log_rr = np.log((a / n1) / (c / n2))
    var_log_rr = (1/a) - (1/n1) + (1/c) - (1/n2)
    se_log_rr = np.sqrt(var_log_rr)
    study_rr = np.exp(log_rr)
    study_lower = np.exp(log_rr - 1.96 * se_log_rr)
    study_upper = np.exp(log_rr + 1.96 * se_log_rr)

    # --- Print individual study estimates ---
    print(f"{'Study':<20} | {'RR':<6} | {'95% CI':<15}")
    print("-" * 45)
    for i in range(len(df)):
        print(f"{study_names[i]:<20} | {study_rr[i]:.3f} | ({study_lower[i]:.3f} - {study_up-
per[i]:.3f})")
    print("-" * 45)

    # 4. Fixed-effects weights calculation
    w_fixed = 1 / var_log_rr

    # 5. Calculate Cochran's Q and I-squared statistic
    weighted_mean_fixed = np.sum(w_fixed * log_rr) / np.sum(w_fixed)
    Q = np.sum(w_fixed * (log_rr - weighted_mean_fixed)**2)
    df_Q = len(df) - 1

    # Truncate I-squared at 0.0 if Q < df_Q
    I2 = max(0.0, (Q - df_Q) / Q) * 100 if Q > df_Q else 0.0
    p_heterogeneity = stats.chi2.sf(Q, df_Q)

    # 6. Calculate Tau-squared (DerSimonian-Laird method for between-study variance)
    if Q > df_Q:
        C = np.sum(w_fixed) - (np.sum(w_fixed**2) / np.sum(w_fixed))
        tau2 = (Q - df_Q) / C
    else:
        tau2 = 0.0

    # 7. Random-effects weights calculation
    w_random = 1 / (var_log_rr + tau2)

    # 8. Calculate pooled random-effects log(RR) and standard error
    pooled_log_rr = np.sum(w_random * log_rr) / np.sum(w_random)
    se_pooled_log_rr = np.sqrt(1 / np.sum(w_random))

    # 9. Convert back to Risk Ratio (RR) and 95% CI
    pooled_rr = np.exp(pooled_log_rr)
    lower_ci = np.exp(pooled_log_rr - 1.96 * se_pooled_log_rr)
    upper_ci = np.exp(pooled_log_rr + 1.96 * se_pooled_log_rr)

    # Calculate p-value for the overall effect
    z_score = pooled_log_rr / se_pooled_log_rr
```

```
    p_effect = 2 * (1 - stats.norm.cdf(abs(z_score)))

    # Print final pooled results
    print(f"Pooled Risk Ratio (RR): {pooled_rr:.3f} (95% CI: {lower_ci:.3f} - {upper_ci:.3f})")
    print(f"P-value for overall effect: {p_effect:.4f}")
    print(f"Heterogeneity: Q = {Q:.2f}, df = {df_Q}, p = {p_heterogeneity:.4f}, I^2 = {I2:.1f}%")
    print(f"Tau^2: {tau2:.4f}\n")

    return pooled_rr, lower_ci, upper_ci, p_effect, I2

# === Create Dataset (N=5) ===
# Events based on Grade 3/4 oral mucositis data
data = {
    'Study': ['Lin (2006)', 'Sangthawan (2013)', 'Ertekin (2004)', 'Watanabe (2010)', 'Sa-
hebnasagh (2023)'],
    'Subgroup': ['Systemic', 'Systemic', 'Systemic', 'Topical', 'Topical'],
    'Zinc_Events': [22, 6, 0, 1, 2],
    'Zinc_Total': [49, 72, 15, 16, 17],
    'Control_Events': [36, 10, 8, 10, 9],
    'Control_Total': [48, 72, 12, 15, 16]
}
df_all = pd.DataFrame(data)

# 1. Run Overall Meta-analysis
calculate_meta_analysis(df_all, "Overall (N=5)")

# 2. Run Systemic Subgroup Meta-analysis
df_systemic = df_all[df_all['Subgroup'] == 'Systemic']
calculate_meta_analysis(df_systemic, "Systemic Zinc Subgroup")

# 3. Run Topical Subgroup Meta-analysis
df_topical = df_all[df_all['Subgroup'] == 'Topical']
calculate_meta_analysis(df_topical, "Topical Zinc Subgroup")
```
